# Designing a Graphene Coating-Based Supercapacitor with Lithium Ion Electrolyte: An Experimental and Computational Study via Multiscale Modeling

**DOI:** 10.3390/nano11112899

**Published:** 2021-10-29

**Authors:** Joseph Paul Baboo, Shumaila Babar, Dhaval Kale, Constantina Lekakou, Giuliano M. Laudone

**Affiliations:** 1 Centre for Engineering Materials, Department of Mechanical Engineering Sciences, Faculty of Engineering and Physical Sciences, University of Surrey, Guildford GU2 7XH, UK; j.baboo@surrey.ac.uk (J.P.B.); shumaila.babar@surrey.ac.uk (S.B.); dk00197@surrey.ac.uk (D.K.); 2 Faculty of Science and Engineering, University of Plymouth, Plymouth PL4 8AA, UK; G.Laudone@plymouth.ac.uk

**Keywords:** supercapacitor, graphene, lithium electrolyte, experimental, simulations

## Abstract

Graphene electrodes are investigated for electrochemical double layer capacitors (EDLCs) with lithium ion electrolyte, the focus being the effect of the pore size distribution (PSD) of electrode with respect to the solvated and desolvated electrolyte ions. Two graphene electrode coatings are examined: a low specific surface area (SSA) xGNP-750 coating and a high SSA coating based on a-MWGO (activated microwave expanded graphene oxide). The study comprises an experimental and a computer modeling part. The experimental part includes fabrication, material characterization and electrochemical testing of an EDLC with xGNP-750 coating electrodes and electrolyte 1M LiPF6 in EC:DMC. The computational part includes simulations of the galvanostatic charge-discharge of each EDLC type, based on a continuum ion transport model taking into account the PSD of electrodes, as well as molecular modeling to determine the parameters of the solvated and desolvated electrolyte ions and their adsorption energies with each type of electrode pore surface material. Predictions, in agreement with the experimental data, yield a specific electrode capacitance of 110 F g^−1^ for xGNP-750 coating electrodes in electrolyte 1M LiPF_6_ in EC:DMC, which is three times higher than that of the high SSA a-MWGO coating electrodes in the same lithium ion electrolyte.

## 1. Introduction

Electrochemical double layer capacitors (EDLCs) are energy storage devices of high power density, high efficiency and long life time while they may utilize low cost materials [1] but are generally of lower energy density than batteries. For many years, there has been extensive research involving electrode materials with small micropores suitable for aqueous electrolytes [2,3] but such electrolytes have the disadvantage of low maximum voltage. Organic electrolytes have extended the voltage window to 3 V [4] with low viscosity solvents, such as acetonitrile (ACN), and have been at the forefront of research and electrode materials development in EDLCs [5]. However, due to the low energy density of EDLCs compared to batteries, hybridized battery-supercapacitor devices [6] have been considered, in addition to lithium-ion capacitors [7,8]. Such devices generally contain a lithium ion electrolyte rather than the traditional organic electrolytes for which EDLC electrode materials have been optimized over many years of research and development in supercapacitors.

Materials of large specific surface area have been favored as electrodes in EDLCs, as it has been thought that they would store a large amount of charge in the form of a monolayer of ions assumed to line their surface according to the Stern layer hypothesis [9,10]. Such electrode materials include activated carbon (AC) in the form of coating [11,12] or fiber mat [13,14], graphene or graphene oxide coatings [15,16,17,18,19] and graphitic and graphene frameworks [20,21,22,23]. Additives are used to increase electrical conductivity, such as carbon black and carbon nanotubes [12,24,25,26] where the latter have also contributed to increased capacitance due to additional surface area [24]. Thermoplastic binders are normally employed for the coating-type electrodes so that they facilitate material recycling [27,28] via dissolution in solvents [29,30]; however, the binder tends to block pores and, hence, reduce the surface area of the porous powder material [24].

A review of the electrode materials performance in different electrolytes revealed that further to the specific surface area of the electrode, pore size is also important. In particular, Huang et al. [31] noticed that electrode materials with pore size close to the electrolyte ion size tend to have the highest specific capacitance. Moreover, it has been remarked that a range of pore sizes is needed, where large pores enhance ion transport so that the small, high capacitance pores are accessible to the ions. As a result, mesoporous electrode materials ensure fast ion transport [32] and yield EDLCs of high power density whereas microporous electrode materials offer high specific capacity and enrich the energy density [33]. It is also well known that size and morphology of particles, as well as how they agglomerate, have also an effect on materials performance [34]. A continuum ion transport model has been developed by our group [35] that takes into account the pore size distribution in each electrode, considering parallel ion transport equations in pores of different size as well as hierarchical interpore ion transport assuming a pore line model. In this model, ions are in either solvated [36] or desolvated form [37], the latter for pores smaller than the solvated ion. This model was used in computer simulations to evaluate the performance of EDLCs with AC electrodes of different pore size distributions and electrolyte 1M LiPF_6_ in EC:EMC: it was found that a peat bog-derived AC coating was better than a phenolic-derived AC coating and a phenolic-derived AC Kynol^®^ fabric, reaching a specific electrode capacitance, C_el,sp_, of 50 F g^−1^ as predicted by a simulated galvanostatic discharge and confirmed by the experimental data [35]. The aim of the current investigation is to increase the capacitance of an EDLC with lithium-ion electrolyte, by considering graphene-based electrodes.

Graphene materials are encountered in a range of specific surface area, SSA_BET_, values from commercial graphitic nanoplatelets of small SSA_BET_ = 150 m^2^ g^−1^ but high electrical conductivity to high surface area graphene of SSA_BET_ of about 3000 m^2^ g^−1^ which is not generally widely available. EDLCs based on XG C-750 GNPs (graphene nanoplatelets) from XG Sciences, of powder SSA_BET_ = 750 m^2^ g^−1^ and coating SSA_BET_ = 500 m^2^ g^−1^ (PVDF binder) [38] exhibited C_el,sp_ = 70 F g^−1^ in electrolyte 1 M TEABF_4_ in ACN [38]. The XG-C-750 GNP powder seems to be of similar SSA_BET_ and atomic composition as the chemically modified graphene (CMG) of Stoller et al.’s work [39] who synthesized the CMG by starting with natural graphite and using a modified Hummer’s method to convert it to graphene oxide (GO) which was reduced to rGO employing hydrazine monohydrate. On the other hand, microwave exfoliated, and reduced graphene oxide (MWGO) offers SSA_BET_ = 2490 m^2^ g^−1^ [16,40] which was further increased to SSA_BET_ = 3100 m^2^ g^−1^ via chemical activation of MWGO with KOH [41]; the latter yielded an EDLC which exhibited C_el,sp_ = 150 F g^−1^ in electrolyte 1 M TEABF_4_ in ACN [41]. Despite the abundance of graphene materials synthesized by various research groups, there is generally lack of research in EDLCs with graphene electrodes and lithium-ion electrolyte while there is a need to research the optimum pore size distribution of graphene electrodes for potential applications of lithium-ion capacitors and hybrid Li-ion battery-supercapacitor devices.

The possibility of adsorption of Li^+^ on a GO surface and further enhancement of capacitance via reversible redox reactions between C=O groups and Li^+^ has been reported and observed as reaction peaks in cyclic voltagrammetry at 3.5 V in charge and 2.3 V in discharge [42]. Further exploration of an rGO EDLC with electrolyte 1M LiPF_6_ in EC:DMC yielded the observation of electrolyte decomposition above 2.8 V of the full EDLC cell (or above 4.5 V vs. Li/Li+ for the positive electrode) [43,44].

The present study investigates graphene-based electrodes for EDLCs with lithium ion electrolyte with the aim to evaluate the effect of pore size distribution (PSD) of the electrode material on the performance of the EDLC. More specifically, the experimental part of the investigation investigates an electrode coating of commercial XG C-750 GNP powder of SSA_BET,powder_ = 750 m^2^ g^−1^. Computational simulations using the continuum ion transport model [35] applied to a pore size distribution are carried out for the galvanostatic charge-discharge of this EDLC as well as an EDLC with a-MWGO (activated MWGO) coating electrodes of high surface area a-MWGO powder of SSA_BET,powder_ = 3100 m^2^ g^−1^ [41], with either organic electrolyte 1M TEABF_4_ in ACN, to compare predictions with experimental data [41], or lithium ion electrolyte. Molecular modeling is employed to determine parameters of the solvated electrolyte ions and the adsorption energy of the desolvated electrolyte ions with each type of electrode material. The continuum model is also updated in terms of a relation for the tortuosity of platelet electrodes. The comparison of the simulation predictions between the two graphene-based electrodes of significantly different SSA (specific surface area) values and PSDs offers an insight into the recommended trends in the design of graphene electrodes in EDLCs with lithium ion electrolyte.

## 2. Materials and Methods

The electrode coating composition was: 85 wt % high purity graphene nanoplatelets from XG Sciences, US (grade C), which will be referred henceforth as xGNP-750 (average surface area: 750 m^2^ g^−1^, average particle diameter: less than 2 μm and bulk density 200–400 kg m^−3^; specified to contain 8.2 at% O and 1.5 at% N from manufacturer’s data), 10 wt% acetylene carbon black (CB, surface area: 75 m^2^ g^−1^, average particle size 42 nm and bulk density: 170–230 kg m^−3^) from Alfa Aesar, US, and 5 wt % polyvinylidene fluoride (PVDF, Mw = 534,000) from Sigma Aldrich, UK. The electrolyte was 1 M LiPF_6_ in EC:DMC 1:1 *v*/*v* from Sigma Aldrich, UK. The separator comprised three layers: two outer layers of Celgard 3501 and a middle layer of Whatman grade GF-F glass fiber filter.

A mixture was prepared for the electrode coating as follows: 12 g of xGNP-750 was weighted in 80 mL of N-methyl-2-pyrrolidone (NMP), the mixture was subjected to sonication in an ultrasound bath for 20 min and further GNP dispersion and exfoliation using OMNI general lab homogenizer (GLH 850) high-shear mixer at 15,000 rpm for 15 min [45]. Then 1.4 g of CB was added to above mixture, subjected to bath sonication (30 min) and high-shear mixing (15 min). Afterwards, 0.7 g of PVDF in 5 mL of NMP solution was added to the above mixture and magnetically stirred for a few hours at 150 °C until a viscous slurry was formed. Finally, the obtained slurry was coated onto the current collector foil using a film applicator and dried in oven at 80 °C overnight to obtain the final electrode. 

The obtained electrodes were cut into 15 mm discs, dried in vacuum oven at 120 °C for 2 h and evacuated in the glove box antechamber overnight in order to remove any solvent/moisture from the electrode surface. Symmetric EDLC cells were fabricated using the xGNP-750 coating electrodes (coating mass of each electrode: 1.9–2 mg), 300 μL lithium ion electrolyte (1 M LiPF_6_ in EC/DMC 1:1 *v*/*v*), and separator.

The surface morphology of the electrodes was characterized using high-resolution scanning electron microscope HR-SEM JEOL-7100 F (JEOL, Belgium). 

The specific surface area (SSA) and pore size distribution (PSD) were determined from nitrogen isotherms from adsorption/desorption experiments in a BELSORP-Max instrument (Microtrac, Japan). GCMC (grand canonical Monte Carlo) simulations were carried out to determine the PSD and the BET (Brunauer–Emmett–Teller) method was applied to determine the SSA (SSA_BET_).

Electrochemical testing of EDLC cells included electrochemical impedance spectroscopy (EIS) in the frequency range of 10 m Hz to 1 M Hz, and galvanostatic charge/discharge (GCD) at various currents (5–50 mA) in the potential range 0 to 2 V.

## 3. Multiscale Modeling

The galvanostatic charge-discharge cycle was modeled using a continuum model presented in [35] which comprises a set of volume-averaged, one-dimensional, ion transport equations (for the cation and the anion) through the EDLC cell thickness (x-direction) catering for different pore sizes from a discretized pore size distribution (PSD) of the porous electrode. In these ion transport equations, the drift current and diffusion terms are preceded by the diffusion coefficient, D_i,p_ of ion i (cation or anion) in pore size p, which is given by the relation [46]:

(1) $$D_{i,p}=\frac{{\delta k}_{B}T}{2\pi \eta \left(d_{\mathrm{solv}.\mathrm{ion},i}{\;\mathrm{or}\;d}_{\mathrm{ion},i}\right)\tau_{p}^{2}}$$

where k_B_ is the Boltzmann’s constant, T is the absolute temperature in Kelvin, δ is the constrictivity factor [35,47], η is the viscosity (function of solute concentrations [35]), τ_p_ is the tortuosity of the porous path of pore size p and d_solv.ion,i_ or d_ion,i_ is the size of the solvated or desolvated ion, respectively, depending on the pore size. 

**Figure 1:**
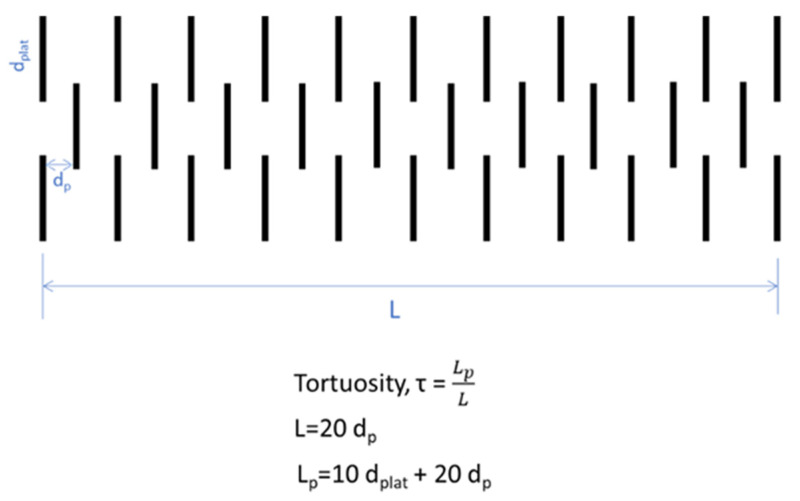
Basic configuration of GNPs to derive the relation for the tortuosity as a function of the geometrical features of the GNP configuration.

Whereas in previous studies the tortuosity was considered as τ_p_ = ε^−0.75^ on the basis of the assumption of spherical electrode particles [46,48], a new relation (2) has been derived for the GNP (graphene nanoplatelet) coating electrodes of this work on the assumption of flat platelets. Figure 1 depicts a basic configuration of staggered GNPs of a lateral dimension d_plat_ and at a distance d_p_ (equal to the slit pore size). The tortuosity is defined as the ratio of the pore path, L_p_, and the shortest path distance, L. Following from Figure 1, the tortuosity, τ_p_, of the pore path between GNPs at a distance of the size of slit pore, d_p_, is given by:

(2) $$\tau_{p}=1+\frac{d_{\mathrm{plat}}}{2d_{p}}\;$$

The dimensions of the solvated and desolvated ions (cation or anion) will be determined on the basis of molecular modeling in Section 4.1. For each pore size of the discretized PSD, if the pore is greater than the solvated ion i, then d_solv.ion,i_ is considered, otherwise d_ion,i_ is considered in Equation (1). However, for pore sizes smaller than the solvated ion, the drift current, diffusion and inter-pore current flux terms are multiplied by a decay factor as in [35], F_decay,i_, given by the equation:

(3) $$F_{\mathrm{decay},i}=e^{-\left(\frac{∆E_{i}}{\mathrm{RT}+E_{\mathrm{EC}}-∆E_{i-\mathrm{pore}}}\right)}$$

where ∆E_i_ is the desolvation energy, R is the ideal gas constant, E_EC_ is the electrochemical energy per mol and ∆E_i-pore_ is the repulsion (positive) or adsorption (negative) energy between ion i and the pore walls, the latter to be determined via molecular simulations in Section 4.1.

Simulations of the GCD cycle were carried out for a symmetric EDLC cell, including numerical calculations in both anode and cathode. The separator was considered a fully permeable membrane of zero thickness. The initial condition determined that all points in the cell were in neutral state and all pores greater than the larger solvated ion were filled with electrolyte at equal concentrations of the positive and negative ion charges, A numerical grid of 100 ∆x spacings (in the x-direction) was used [35,49,50,51,52], comprising 50 spacings along the cathode thickness and 50 spacings along the anode thickness.

## 4. Results and Discussion

### 4.1. Molecular Modeling

**Figure 2:**
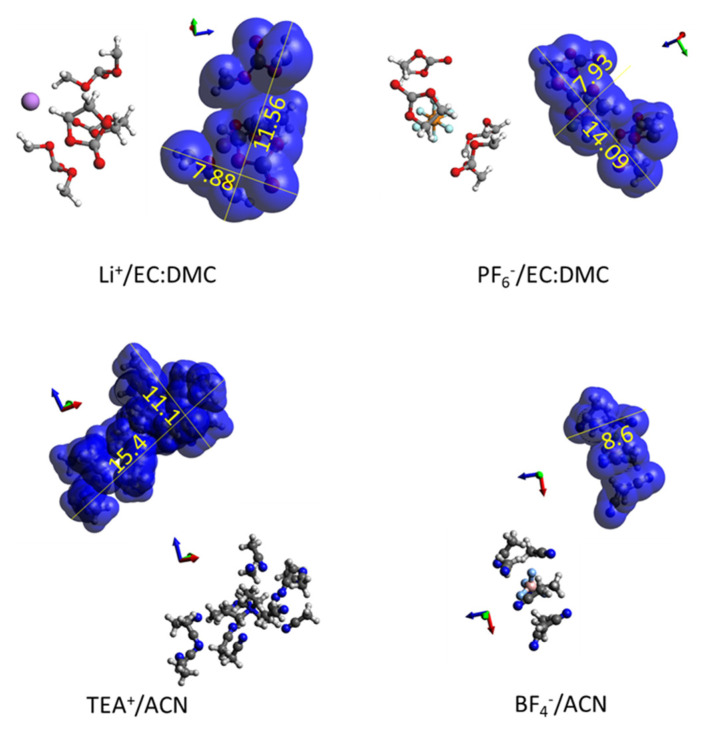
Solvated ions of electrolytes LiPF_6_/EC:DMC and TEABF_4_/ACN in ball-and-stick representation and also wrapped with the van der Waals surface (or Connolly surface).

The coordination and binding energies of each ion, Li^+^ and PF6^-^, with each solvent separately were determined using the amorphous cell of Materials Studio v4.1, which was first optimized geometrically using COMPASS and then subjected to an MD (molecular dynamics) simulation. This yielded the results reported in Table 1. Tenney and Cygan [53] carried out MD simulations for 1 M LiPF_6_ in EC:DMC and found out that n_Li+/EC:DMC_ = 3.2 consisting of 1.6 EC and 1.6 DMC molecules. On the other hand, MD simulations of LiPF_6_ in EC:DMC near a graphite electrode surface yielded n_Li+/EC:DMC:PF6_ = 5 at zero charge, consisting of 2 EC, 2 DMC and 1 PF_6_^−^ ion, and n_Li+/EC:DMC_ = 4 when the graphite surface was charged consisting of 3 EC and 1 DMC molecules [54]. In the present study, geometrical optimization simulations in Materials Studio yielded minimum energy configurations for n_Li+/EC:DMC_ = 4 consisting of 2 EC and 2 DMC molecules and also n_PF6-/EC:DMC_ = 4 also consisting of 2 EC and 2 DMC molecules. The final solvated ion structures are presented in Figure 2. The van der Waals surface model (or Connolly surface), was used to derive the minimum and maximum dimensions of the solvated ions. With regards to desolvation energies, there is a large variation of values reported in the literature for Li^+^/EC:DMC, ranging from +4 to −41.4 kJ mol^−1^ [55] to −121 kJ mol^−1^ [56], while it is known that the solvated PF_6_^−^ ion in is easily desolvated in organic solvents. As the values derived from simulations in this study and presented in Table 1 are in the range of values presented in the literature, for consistency we shall use our values in Table 1 as input data in the simulations in Section 4.4.

GCD simulations in this study were also performed for a second electrolyte, TEABF_4_ in acetonitrile, for which coordination numbers and desolvation energy values have been determined in the literature [57]. Molecular modeling including geometrical optimization using Materials Studio was carried out for this electrolyte for both desolvated and solvated ions with values of the coordination numbers from [57]; The results are included in Table SI-1 regarding electrolyte input data. Figure 2 presents the solvated ions TEA^+^/ACN and BF_4_^−^/ACN and illustrates the determination of their dimensions from the van der Waals surface.

**Figure 3:**
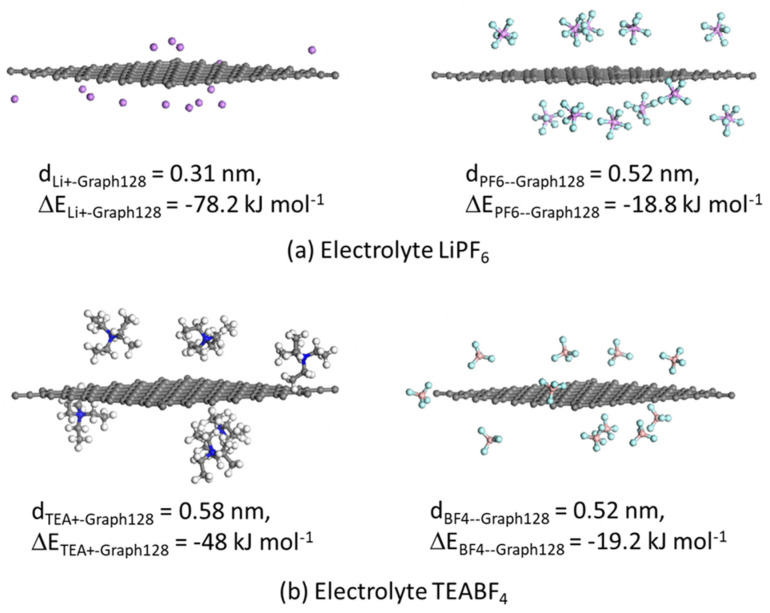
Graphite sheet with 128 C atoms, Graph128 (representing a-MWGO in this study), coordinated with either the desolvated cation or the desolvated anion of electrolyte (**a**) LiPF_6_ and (**b**) TEABF_4_; noted the average distance and the adsorption energy between Graph128 and the center of ion mass.

The next issue to address is about slit pores smaller than the minimum size of each solvated ion, which happens to be 0.79 nm for both cation and anion in LiPF_6_ in EC:DMC, as can be seen in Figure 2, and 1.11 nm for TEA^+^/ACN and 0.86 nm for BF_4_^−^/ACN as can be seen in Table SI-1. In this case, the Blends Module in Materials Studio was utilized to determine the adsorption energy of the desolvated ions TEA^+^ and BF_4_^−^ with a graphite sheet (for the simulations of the GCD cycle of a-MWGO EDLC) and the adsorption energy of Li^+^ and PF_6_^−^ ions with the xGNP-750 type of graphitic sheet. The Blends Module uses the Monte Carlo method for sampling of configurations [58] where simulations in the present study for the calculation of adsorption energy employed 107 samples and a reference temperature of 298 K. A graphite sheet of 128 carbon atoms was built and subjected to geometrical optimization. Figure 3 displays the assemblies of Graph128 with the cations and anions for each of the electrolytes: (a) LiPF_6_ and (b) TEABF_4_. In general, ion adsorption is observed, with the cations exhibiting higher adsorption energy given the relative electronegativity of graphene; in particular, Li^+^ demonstrates a higher adsorption energy than TEA^+^. The distance between Li^+^ and Graph128 is also the smallest, compared to the other ions which is due to the small size of the lithium ion as well as the corresponding high adsorption energy.

**Figure 4:**
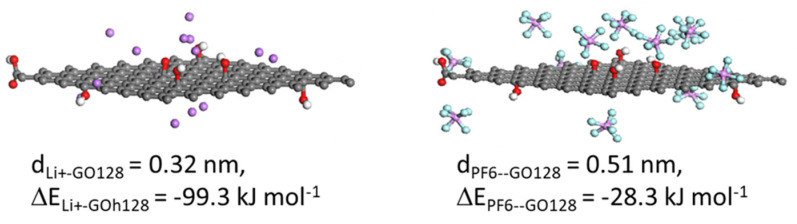
Graphitic oxide sheet with 128 C atoms, GO128 (representing xGNP-750 in this study), coordinated with either the desolvated cation or the desolvated the anion of electrolyte LiPF_6_; noted the average distance and the adsorption energy between GO128 and the center of ion mass.

With regards to the xGNP-750 material used in the experimental part of this study, it contains 8.2 at% O (according to manufacturer’s data). The particular xGNP grade C powder (SSA_BET_ = 750 m^2^ g^−1^) employed in this study was characterized by XPS [59,60], which showed C–O (at 285.5 eV) and C=O (at 288.4 eV) groups at atomic ratio 3:1, respectively [59], while the FTIR spectrum showed a prominent -OH peak and a small C=O peak [59]. Hence, for the xGNP-750 layer of this study, the following groups were introduced in the Graph128 model converting it to GO128: 5 at% C–OH, 1 at% C-O-C and 2 at% C=O on the basal plane, and 1.15 at% COOH at corners. Figure 4 displays the assemblies of GO128 with the desolvated cation and anion of electrolyte LiPF_6_: it can be seen that the resulted values of the adsorption energies between GO128 and each ion are higher than those with Graph128, demonstrating the binding effect of the O functional groups. The average distances between each ion and GO128 are slightly higher than for Graph128 to be able to accommodate the O functional groups in the case of GO128.

All electrolyte parameter values presented in Table SI-1 are used as input data in the continuum model simulations of the EDLC in Section 4.4. Furthermore, Table SI-2 presents input data for the electrodes xGNP-750 and a-MWGO [10] for the continuum simulations in Section 4.4.

### 4.2. Electrode Characterization

**Figure 5:**
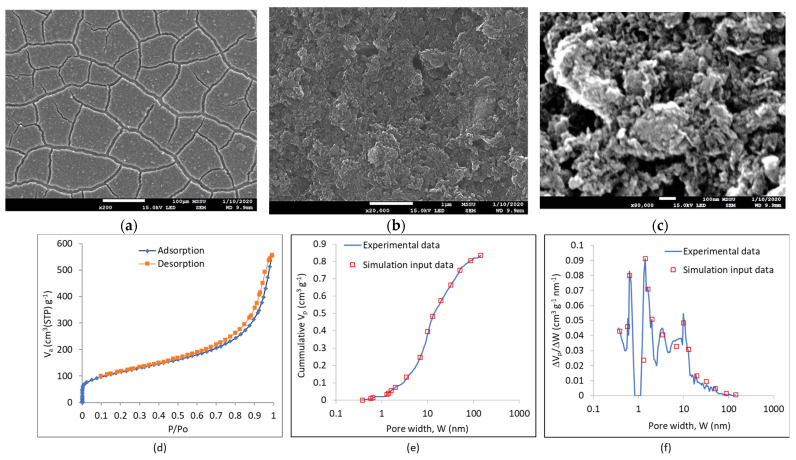
Results from the characterisation of electrode coating. (**a**–**c**) SEM images of the electrode coating at three different magnifications, sequentially: scale bar: 100 μm, 1 μm, 100 nm; (**d**–**f**): results of the nitrogen adsorption/desorption tests for the characterization of the electrode coating: (**d**) experimental data of adsorption/desorption isotherms; (**e**) cumulative pore size distribution graph; (**f**) incremental pore size distribution graph.

Figure 5a–c present SEM images of the electrode coating at three different magnifications. GNPs can be seen in Figure 5b of lateral dimensions generally in the range of 100–500 nm. This is in agreement to characterization data from the study by Chong et al. [61] for the same xGNP-750 material, in which a particle diameter in the range of 50–600 nm, with a mean diameter at 200 nm, was obtained by laser light scattering and 300 nm by SEM imaging. Due to the small lateral dimensions of the xGNP-750 platelets, there is no extensive GNP folding and curling usually observed in large nanoplatelets [40]. This means that the GNPs are well packed in the electrode coating with small interparticle spaces of about 200 nm, as can be seen in Figure 5c. The low magnification SEM image in Figure 5a reveals a network of cracks in the electrode coating, with coating islands of about 100 μm diameter and crack channel width of 10–20 μm. These channels width will be inputted as the macropore reservoir width in the computational model runs, considering a rectangular cross-section macropore.

Figure 5d presents the adsorption/desorption isotherms for the electrode coating. The isotherms show a large amount of multilayer adsorption and a hint of hysteresis (mesoporosity with capillary condensation taking place). The PSDs derived from the experimental data via GCMC simulations in Figure 5e,f exhibit a complex structure with multiple peaks as displayed in Figure 5f which is different from previous activated carbon (AC) -based coatings examined by our group [35]: AC-based electrode coatings had shown bimodal pore size distribution for peat bog-derived charcoal with a main peak at 0.635 nm and a smaller peak at 1.41 nm (SSA_BET_ = 808.3 m^2^ g^−1^), and a broad monomodal distribution for phenolic-derived AC with a main peak at 1.3 nm and a range from 0.5 to 3.5 nm (SSA_BET_ = 1273.7 m^2^ g^−1^). The xGNP-750-based coating in this study exhibits peaks at 0.57, 0.635, 1.61, 1.97, 3.46, 9.93, 12.83 nm and small distinct peaks from 14 to 145 nm. The specific surface area of the GNP-based coating is SSA_BET_ = 410 m^2^ g^−1^, which is much smaller than that of the AC-based coatings of our previous studies [35].

A discrete PSD was fitted to the experimental line, in order to use the discrete PSD as input data for the simulations using the continuum model outlined in Section 3. The fitted discrete PSDs consisted of 17 pore sizes fitting the N_2_ adsorption data in Figure 5e,f and an additional macropore of 15 μm representing the crack macrochannels in the electrode coating as shown in Figure 5a (top image). Table SI-2a presents the data for the discretized PSD of xGNP-750 electrode coating. In the process of evaluation of the most suitable graphene-based electrode for an EDLC with lithium-ion electrolyte, a large specific surface area a-MWGO electrode was considered in the continuum model simulations in Section 4.4, where experimental data was obtained from [41]. In particular, the PSD of a-MWGO was processed and discretized to obtain suitable input data for the simulated PSD of the a-MWGO coating in Section 4.4, and the derived data of the discretized PSD is given in Table SI-2b: the dataset comprises 77 pore sizes to accurately represent the PSD of a-MWGO [41] with more multiple peaks than the PSD of xGNP-750.

### 4.3. Results of the Electrochemical Testing

**Figure 6:**
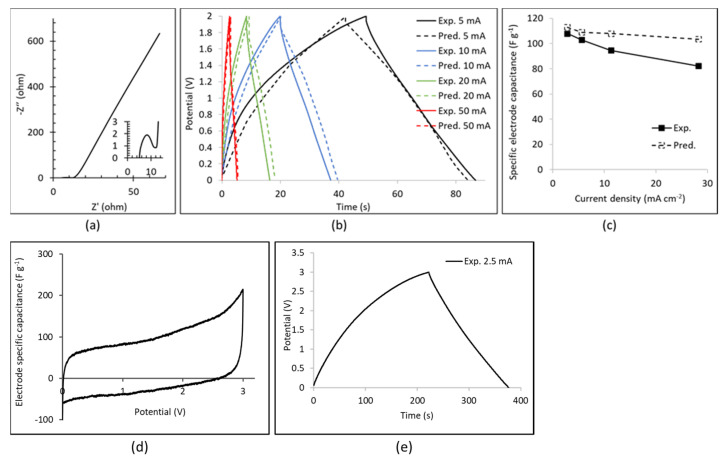
Results of the experimental study and computer simulation for the symmetric EDLC of 1.77 cm^2^ with xGNP-750 composite coating electrodes and electrolyte 1M LiPF_6_ in EC:DMC. (**a**) Nyquist impedance plot from the EIS test data; (**b**) GCD curves at different currents: experimental data and simulation predictions; (**c**) Plot of specific electrode capacitance versus current density: experimental data and simulation predictions; (**d**) CV test plot in the range of 0–3 V at 5 mV s^−1^; (**e**) GCD test plot in the range of 0–3 V at 2.5 mA.

A symmetric EDLC was tested (cell of area of 1.77 cm^2^), with xGNP-750 coating electrodes and electrolyte 1M LiPF_6_ in EC:DMC. Figure 6a presents the Nyquist impedance plot from the EIS test. From the insert it can be seen that the first intercept on the real impedance, Z’, axis is R1 = 5 ohm which represents the electrolyte, electrode and separator resistance. The diameter of the semicircle, i.e., the difference between the second and first intercept on the Z’ axis, is R2 = 6.87 ohm which represents the sum of contact resistances mainly between electrode and current collector. The main impedance line is straight for frequencies below 50 Hz (knee point) with a loss tangent tan δ = 0.1.

Figure 6b presents the experimental results of the GCD tests in the range of 0–2 V at currents I = 5, 10, 20 and 50 mA, from the discharge part of which the electrode specific capacitance, C_el,sp_, is determined according to the equation:

(4) $$C_{\mathrm{el},\mathrm{sp}}=\frac{4{\mathrm{It}}_{\mathrm{discharge}}}{V_{\max}\left(2m_{\mathrm{el}}\right)}$$

where t_discharge_ is the total discharge time, V_max_ is the maximum voltage after the voltage drop at the start of discharge and m_el_ is the mass of each electrode coating. The electrode specific capacitance values versus current density are presented in Figure 6c where it can be seen that 108 F g^−1^ is reached at 2.82 mA cm^−2^. This value is 2.72 times higher than the corresponding C_el,sp_ of an AC-coating symmetric EDLC with electrolyte 1M LiPF_6_ in EC:EMC, where the latter demonstrated C_el,sp_ = 39.7 F g^−1^ at 2.82 mA cm^−2^ [35]. Further tests were carried out to higher voltages in the range of 0–3 V and the experimental data are presented in Figure 6d,e for the CV and the GCD test, respectively. The CV test exhibits some parasitic reactions above 2.8 V, which is consistent with observations in the literature of electrolyte decomposition above 2.8 V for the full EDLC cell (or above 4.5 V vs. Li/Li+ for the positive electrode) [43,44]. The GCD test at 2.5 mA to a maximum potential of 3 V also demonstrates this effect during charge, although it maintains the high discharge capacitance calculated in Figure 6b. 

### 4.4. Results of Computational Simulations Based on Continuum Ion Tranport Model

Computer simulations of the GCD cycle of the EDLC of the experimental Section 4.3 were performed using the continuum model presented in [35,62] with the updates outlined in Section 3. The predicted GCD curves are displayed in Figure 6b together with the corresponding experimental curves. There seems to be relatively good agreement between predictions and experimental data, with generally longer predicted GCD cycles. Figure 6c depicts the predicted specific electrode capacitance versus the current density from the galvanostatic discharge predictions. Although the predicted C_el,sp_ value is only 4.6% higher than the experimental value at the low current density of 2.82 mA cm^−2^, it is 25.9% higher at the high current density of 28.2 mA cm^−2^. The same trend between predictions and experimental data was observed for the AC-based EDLC in our previous study [35], where the continuum model simulations were based on an inputted discretized PSD of 15 pore sizes in the electrode. At such high current densities, ion transport through meso- and macropores dominates, and charge is stored at the wall surface of such meso- and macropores. Therefore, accurate values in the inputted PSD in the meso- and macropore range are critical for accurate predictions at high current density. Nitrogen adsorption experiments were conducted on powdered electrode coating, i.e., scraped and powdered coating material in the present study and also in our previous study [35], which means that some of the macropore peaks in the coating were not detected in the PSD in Figure 5f, which might explain the disagreement between capacity predictions and experiment in Figure 6c. 

**Figure 7:**
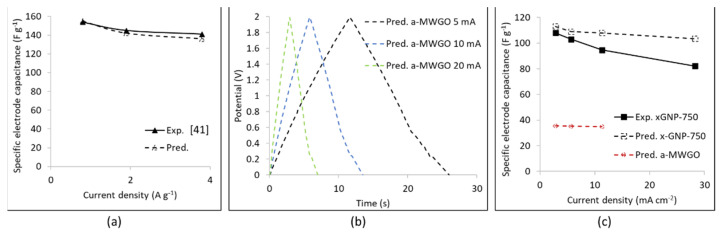
Results of the computer simulations of GCD cycles of a symmetric EDLC with a-MWGO electrodes: (**a**) electrolyte 1M TEABF_4_ in ACN: plot of the specific electrode capacitance versus current density and comparison between predictions and capacitance values calculated from the experimental charge-discharge data of [41]; (**b**) electrolyte 1M LiPF_6_ in EC:DMC: predicted GCD curves; (**c**) electrolyte 1M LiPF_6_ in EC:DMC: plot of the specific electrode capacitance versus current density and comparison between a-MWGO electrode (predictions) and xGNP-750 electrode (predictions and experimental data).

A large specific surface area electrode material was investigated in the computer simulations, a-MWGO from the experimental study of Zhu et al. [41]. Experimental data exist [41] from the electrochemical testing of a symmetric EDLC with this electrode material and electrolyte 1M TEABF_4_ in ACN. Hence, simulations were carried out first for an EDLC with electrolyte 1M TEABF_4_ in ACN with the electrolyte input data from Table SI-1 and electrode input data from Table SI-2b. Figure 7a depicts the predicted specific electrode capacitance versus the current density, where very good agreement can be seen against the corresponding experimental data [41] which might be attributed to the large number of inputted points of the discretized PSD (77 pore sizes) for the a-MWGO electrode (Table SI-2b) and generally the accuracy of the inputted PSD.

The next step was a computer simulation of the GCD cycle of a symmetric EDLC with a-MWGO coating electrodes and electrolyte 1M LiPF_6_ in EC:DMC, where the EDLC was a coin cell of area 1.77 cm^2^ as in the case of the EDLC in Figure 6. The predicted GCD curves are presented in Figure 7b: when compared to Figure 6b, it is immediately clear that the GCD cycles of the a-MWGO EDLC are much shorter than the corresponding GCD cycles of the xGNP-750 EDLC at the same currents. Furthermore, Figure 7c displays the plot of the specific electrode capacitance versus current density during discharge, where it is evident that the predicted C_el,sp_ values for the a-MWGO EDLC with electrolyte 1M LiPF_6_ in EC:DMC are about 30% the values of the xGNP-750 EDLC with the same electrolyte. This is an unexpected result, given that the a-MWGO powder has a specific surface area of 3100 m^2^ g^−1^ against SSA_BET_ = 750 m^2^ g^−1^ for the xGNP-750 powder. On the other hand, the a-MWGO based EDLC has a much higher C_el,sp_ in the electrolyte 1M TEABF_4_ in ACN than in 1M LiPF_6_ in EC:DMC. Table SI-1 shows that both solvated and desolvated ions are smaller for LiPF_6_ in EC:DMC than in TEABF_4_ in ACN, which means that the cations are mostly in “bulk” transport mode in the a-MWGO electrode, especially for the large surface area peak at 3.8 nm pore size, rather than lining the pore walls whereas the larger cations of TEABF_4_ move slower and may have a better chance to be attracted in the Stern layer of charge storage. On the contrary, xGNP-750 has two high surface area peaks at 0.62 nm and 1.36 nm pore sizes, which offers a better opportunity to trap the transported cations of LiPF_6_ in the Stern layer and increase the charge storage. Moreover, xGNP-750 offers higher adsorption energy for the cation and anion of LiPF_6_ (Figure 4) than a-MWGO (Figure 3a), therefore it leads to faster ion desolvation and ingress of desolvated ions into small micropores, which would further enhance the electrode capacitance.

**Figure 8:**
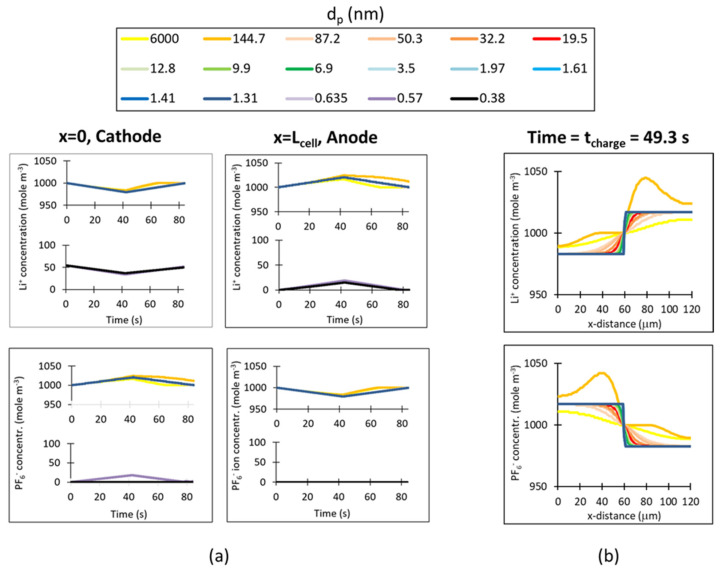
Ion concentration in different pore sizes from the electrode PSD, predicted by the computer simulation of the GCD cycle of the xGNP-750 EDLC with electrolyte 1M LiPF_6_ in EC:DMC; (**a**) ion concentration evolution during charge and discharge, at x = 0 (in the cathode by the current collector) and at x = L_cell_ (in the anode by the current collector); (**b**) ion concentration profiles at the end of charge, from x = 0 through the cathode and anode to x = L_cell_.

Figure 8a illustrates the evolution of the Li^+^ and PF_6_^−^ ion concentration during the charge and discharge at the EDLC electrode edges, by the current collector. It is evident that ion transport progresses via an ion exchange mechanism. During charge the Li^+^ concentration falls in the cathode by 20 mol m^−3^ in the macro-, meso- and micropores that can accommodate the solvated cations and by 18 mol m^−3^ in the small micropores that can only accommodate desolvated cations; on the other hand, Li+ concentration increases in the anode during charge by 20 mol m^−3^ of solvated cations in the macro-, meso- and micropores and by 15 mol m^−3^ of desolvated cations in the small micropores. The concentration of PF_6_^−^ anions follows the opposite pattern: increasing in the cathode and falling in the anode. Figure 8b displays the ion concentration profiles through the cell from the cathode current collector border (x = 0) to the anode current collector border (x = L_cell_ = 120 μm) at the end of the charge, where the separator is located in the middle, at x = 0.5 L_cell_ = 60 μm. It can be seen that the Li^+^ concentration experiences a step increase in the anode and the PF_6_^−^ concentration experiences a step increase in the cathode. In most pores the step change in the concentration profile occurs smoothly through the separator, apart from the macropore of 144.7 nm in which there is still a concentration wave in the profile of both ions.

## 5. Conclusions

The present study has investigated the effect of PSD and specific surface area of graphene electrodes in EDLCs with a lithium ion electrolyte 1 M LiPF_6_ in EC:EMC 50:50 v/v. The update in the tortuosity relation proposed by relation (2) and Figure 1 in Section 3 was crucial in yielding lower diffusion coefficient values due to the high tortuosity of GNPs compared to AC particle coating electrodes and was beneficial for the agreement between predictions and experimental data in the case of xGNP-750 coating electrodes and lithium ion electrolyte in Figure 6b and a-MWGO coating electrodes and 1M TEABF_4_ in ACN electrolyte in Figure 7a. 

Molecular modeling indicated smaller size for the solvated and desolvated Li^+^ ions in EC:DMC solvents against the corresponding TEA^+^ ions in ACN, meaning that the former could ingress in smaller electrode pores than TEA^+^. This resulted in higher specific electrode capacitance of xGNP-750 in 1M LiPF_6_ in EC:DMC, C_el,sp_=110 F g^−1^ in both experimental data and predictions of this study, than in 1 M TEABF_4_ in ACN, C_el,sp_ = 70 F g^−1^ in [38]. The PSD of the xGNP-750 coating exhibited multiple peaks in the range of 0.4 to 10 nm, compared to the bimodal PSD of AC coating [35], which seemed to favor a high specific electrode capacitance, C_el,sp_ = 110 F g^−1^, in the xGNP-750 EDLC with 1M LiPF_6_ in EC:DMC of this study, against C_el,sp_ = 40 F g^−1^, for AC coating electrode [35]. Molecular simulations also indicated higher adsorption energy for the Li^+^ and PF_6_^−^ ions by oxygen-containing functional groups in xGNP-750 (Figure 4) than by a plain graphene or graphitic surface (Figure 3a), which would facilitate desolvation and ingress of desolvated LiPF_6_ electrolyte ions in small micropores of xGNP-750 in comparison with AC and a-MWGO pores the surface of which contain much smaller amount of O-groups, and, hence, they would increase the specific capacitance of xGNP-750 in lithium ion electrolyte. Therefore, the xGNP-750 coating is highly recommended as electrode in EDLCs with lithium ion electrolyte.

The a-MWGO coating has more than 4 times higher specific surface area than the xGNP-750 coating and a PSD peak at the pore size of 9.65 nm [41]; the latter seems to facilitate transport of the large solvated TEA^+^/ACN ions to the high surface area micropores, resulting in high specific capacitance, C_el,sp_ = 155 F g^−1^, for electrolyte 1M TEABF_4_ in ACN, as predicted in this study and in agreement with the experimental data in [41]. However, very low specific electrode capacitance, C_el,sp_ = 35.5 F g^−1^, was predicted for electrolyte 1M LiPF_6_ in EC:DMC, which means that the a-MWGO electrode is not recommended for the lithium ion electrolyte (even if it has high surface area), as its PSD includes many large pore size peaks involved in the “bulk transport” of the small electrolyte ions rather than in their adsorption in the charge storage layer.

## Figures and Tables

**Table 1:** Parameters of the electrolyte ions in different solvents as determined from molecular simulations.

	Li^+^/EC	Li^+^/DMC	PF_6_^−^/EC	PF_6_^−^/DMC
Coordination number, n_i_	3.35	2.75	7	5.15
Desolvation energy, E_i_ (kJ mol^−1^)	−78.26	−52.93	−6.6	−6.76

## Data Availability

Data will be provided after reasonable requests.

## Supplementary Materials

The following are available online at https://www.mdpi.com/article/10.3390/nano11112899/s1, Table SI-1: Values of parameters and properties of the electrolyte system used as input data in the continuum simulation in Section 4.4, Table SI-2: Input data for the electrodes xGNP-750 and a-MWGO [10] for the continuum simulations in Section 4.4.
