# Supplementary material for: Designing a Graphene Coating-Based Supercapacitor with Lithium Ion Electrolyte: An Experimental and Computational Study via Multiscale Modeling
Source: Nanomaterials (Basel). 2021 Oct 29;11(11):2899. doi: 10.3390/nano11112899 (PMC8623662; doi:10.3390/nano11112899)
Supplement: Supplementary file 1 [file nanomaterials-11-02899-s001.zip › nanomaterials-1392574-supplementary.pdf]

Designing a Graphene Coating-Based Supercapacitor with Lithium Ion Electrolyte: An Experimental and Computational Study via Multiscale Modeling

Joseph Paul Baboo [1], Shumaila Babar [1], Dhaval Kale [1], Constantina Lekakou [1,*] and Giuliano M. Laudone [2]

[1] Centre for Engineering Materials, Department of Mechanical Engineering Sciences, Faculty of Engineering and Physical Sciences, University of Surrey, Guildford GU2 7XH, UK; j.baboo@surrey.ac.uk (J.P.B.); shumaila.babar@surrey.ac.uk (S.B.); dk00197@surrey.ac.uk (D.K.)
[2] Faculty of Science and Engineering, University of Plymouth, Plymouth PL4 8AA, UK; G.Laudone@plymouth.ac.uk
* Correspondence: c.lekakou@surrey.ac.uk

SIMULATION INPUT DATA

Table SI-1. Values of parameters and properties of the electrolyte system used as input data in the continuum simulation in section 4.4

**Electrolyte**	**1 M $LiPF_6$ /EC:DMC 50:50 v/v**	**Electrolyte**	**1 M $TEABF_4$/ACN**
Solvent viscosity η_o (mPa s)	0.88 [1]	Solvent viscosity η_o (mPa s)	0.34 [6]
Solvent dielectric constant, ε_r	41 taken as average of interpolated values from [2] and [3]	Solvent dielectric constant, ε_r	36 [7]
Desolvated Li^+<br>d_{Li+} (nm)<br>$d_{Li+,min}$ (nm)<br>$d_{Li+,max}$ (nm)<br>V_{Li+} (nm^3)	 0.18<br>0.18<br>0.18<br>0.00305	Desolvated TEA^+<br>d_{TEA+} (nm)<br>$d_{TEA+,min}$ (nm)<br>$d_{TEA+,max}$ (nm)<br>V_{TEA+} (nm^3)	 0.706<br>0.706<br>1<br>0.261
Adsorption energy of desolvated Li^+:<br>$\Delta E_{Li+/xGNP\ slit\ pore}$<br>(kJ mol^{-1})	-99.26	Adsorption energy of desolvated TEA^+:<br>$\Delta E_{TEA+/a\text{-}MWGO\ slit\ pore}$<br>(kJ mol^{-1})	-48
Desolvated PF_6^-<br>d_{PF6-}(nm)<br>$d_{PF6-,min}$ (nm)<br>$d_{PF6-,max}$ (nm)<br>V_{PF6-} (nm^3)	 0.5<br>0.5<br>0.5<br>0.0654	Desolvated BF_4^-<br>d_{BF4-}(nm)<br>$d_{BF4-,min}$ (nm)<br>$d_{BF4-,max}$ (nm)<br>V_{BF4-} (nm^3)	 0.46<br>0.46<br>0.46<br>0.051
Adsorption energy for desolvated PF_6^-:<br>$\Delta E\ _{PF6/xGNP\ slit\ pore}$<br>(kJ mol^{-1})	-28.3	Adsorption energy for desolvated BF_4^-:<br>$\Delta E\ _{BF4/a\text{-}MWGO\ slit\ pore}$<br>(kJ mol^{-1})	-19.2
Solvated Li^+/EC:DMC:<br>$d_{Li+/EC:DMC}$ (nm)<br>$d_{Li+/EC:DMC,min}$ (nm)<br>$d_{Li+/EC:DMC,max}$ (nm)	 0.79<br>0.79<br>1.16	Solvated TEA^+/ACN<br>d $_{TEA+/ACN}$ (nm)<br>d $_{TEA+/ACN,min}$ (nm)<br>d $_{TEA+/ACN,max}$ (nm)	 1.11<br>1.11<br>1.54

n $_{Li+/EC:DMC}$	4	n $_{TEA+/ACN}$	15.5 [8]
$E_{Li+/EC:DMC}$ (kJ/mol)	-65.6	$E_{TEA+/ACN}$ (kJ/mol)	-222 [8]
Desolvation:<br>Δn $_{Li+/EC:DMC}$	4	Desolvation:<br>Δn $_{TEA+/ACN}$	9.5 [8]
Solvated PF_6/EC:DMC:<br>d $_{PF6-/EC:DMC}$ (nm)<br>=$d_{PF6-/EC:DMC,min}$<br>=$d_{PF6-/EC:DMC,max}$	 <br>0.79<br>0.79<br>1.41	Solvated BF_4/ACN:<br>$d_{BF4-/ACN}$ (nm)<br>=$d_{BF4-/ACN,min}$<br>=$d_{BF4-/ACN,max}$	 <br>0.86<br>0.86<br>1.45
n $_{PF6-/EC:DMC}$	4	$n_{BF4-/ACN}$	6.75 [8]
$E_{PF6-/EC:DMC}$ (kJ/mol)	-6.7	$E_{BF4-/ACN}$ (kJ/mol)	-196 [8]
Δn $_{PF6-/EC:DMC}$	4	$\Delta n_{BF4-/ACN}$	3.4 [8]
Conductivity<br>σ_2 (S m^{-1})	1.14 [4-5]	Conductivity<br>σ_2 (S m^{-1})	6 [9]

Table SI-2. Input data for the electrodes xGNP-750 and a-MWGO [10] for the continuum simulations in section 4.4

Table SI-2a. Input data for the electrode xGNP-750; coating thickness = 60 μm; coating areal density = 1.10 mg cm^{-2} (from the experimental part of this study)

**No**	**1**	**2**	**3**	**4**	**5**	**6**	**7**	**8**
**d_p (nm)**	15x10^3	144.66	87.23	50.32	32.16	19.52	12.83	9.93
**ΔV_p (cm^3 g^{-1})**	2.127	0.03055	0.05415	0.0857	0.1815	0.0891	0.0892	0.1476
**No**	**9**	**10**	**11**	**12**	**13**	**14**	**15**	**16**
**d_p (nm)**	6.89	3.46	1.97	1.61	1.41	1.31	0.635	0.5725
**ΔV_p (cm^3 g^{-1})**	0.1125	0.059951	0.018438	0.0142	0.00913	0.01887	0.00515	0.007267
**No**	**17**							
**d_p (nm)**	0.3788							
**ΔV_p (cm^3 g^{-1})**	0.00054							

Table SI-2b. Input data for the electrode a-MWGO, derived from the processing and discretisation of the PSD in [10]; coating thickness = 60 μm; coating areal density = 1.25 mg cm^{-2} [10]

**No**	**1**	**2**	**3**	**4**	**5**	**6**	**7**	**8**
**d_p (nm)**	500	39.99	33.44	24.45	12.6	10.9	9.46	8.65
**ΔV_p (cm^3 g^{-1})**	13.21	0.01018	0.0045	0.0032	0.0038	0.003	0.0045	0.0056
**No**	**9**	**10**	**11**	**12**	**13**	**14**	**15**	**16**
**d_p (nm)**	8.2	7.65	7.07	6.84	6.39	6.11	5.85	5.72
**ΔV_p (cm^3 g^{-1})**	0.0072	0.0099	0.0125	0.015	0.022	0.024	0.028	0.032
**No**	**17**	**18**	**19**	**20**	**21**	**22**	**23**	**24**
**d_p (nm)**	5.47	5.35	5.17	4.835	4.73	4.68	4.42	4.135
**ΔV_p (cm^3 g^{-1})**	0.0362	0.041	0.0434	0.0449	0.0478	0.051	0.055	0.0555
**No**	**25**	**26**	**27**	**28**	**29**	**30**	**31**	**32**
**d_p (nm)**	3.95	3.82	3.7	3.58	3.5	3.3	3.2	3.13
**ΔV_p (cm^3 g^{-1})**	0.1164	0.1130	0.1040	0.0938	0.0818	0.0688	0.0582	0.0439
**No**	**33**	**34**	**35**	**36**	**37**	**38**	**39**	**40**
**d_p (nm)**	2.89	2.86	2.64	2.45	2.34	2.16	2.04	1.96
**ΔV_p (cm^3 g^{-1})**	0.0375	0.0395	0.0387	0.0341	0.0304	0.0265	0.0227	0.024
**No**	**41**	**42**	**43**	**44**	**45**	**46**	**47**	**48**
**d_p (nm)**	1.89	1.77	1.73	1.55	1.54	1.5	1.45	1.38
**ΔV_p (cm^3 g^{-1})**	0.0247	0.0248	0.0255	0.0243	0.0233	0.0265	0.0235	0.0215
**No**	**49**	**50**	**51**	**52**	**53**	**54**	**55**	**56**
**d_p (nm)**	1.32	1.28	1.24	1.18	1.11	1.07	1.01	0.99
**ΔV_p (cm^3 g^{-1})**	0.0189	0.0154	0.011	0.0086	0.0055	0.0062	0.0069	0.0078
**No**	**57**	**58**	**59**	**60**	**61**	**62**	**63**	**64**
**d_p (nm)**	0.98	0.935	0.89	0.855	0.82	0.78	0.75	0.72
**ΔV_p (cm^3 g^{-1})**	0.0089	0.0105	0.0152	0.0151	0.0158	0.0132	0.01289	0.0103
**No**	**65**	**66**	**67**	**68**	**69**	**70**	**71**	**72**
**d_p (nm)**	0.67	0.65	0.64	0.60	0.57	0.55	0.52	0.50
**ΔV_p (cm^3 g^{-1})**	0.0108	0.0111	0.0147	0.0171	0.0152	0.0136	0.0125	0.0099
**No**	**73**	**74**	**75**	**76**	**77**			
**d_p (nm)**	0.48	0.46	0.44	0.42	0.40			
**ΔV_p (cm^3 g^{-1})**	0.0065	0.0042	0.0030	0.00135	0.0007			

REFERENCES

[1] M. Dahbi, F. Ghamouss, F. Tran-Van, D. Lemordant, M. Anouti, Comparative study of EC/DMC LiTFSI and LiPF6 electrolytes for electrochemical storage. Journal of Power Sources 196 (2011) 9743– 9750.

[2] I.N. Daniels, Z. Wang, B.B. Laird, Dielectric properties of organic solvents in an electric field. J. Phys. Chem. C 121 (2017) 1025–1031.

[3] E.R. Logan, E.M. Tonita, K. L.Gering, L. Ma, M.K.G. Bauer, J. Li, L.Y. Beaulieu, J.R. Dahn, A study of the transport properties of ethylene carbonate-free Li electrolytes. Journal of The Electrochemical Society 165 (2018) A705-A716.

[4] C.L. Berhaut, D. Lemordant, P. Porion, L. Timperman, G.Schmidt, M. Anouti, Ionic association analysis of LiTDI, LiFSI and $LiPF_6$ in EC/DMC for better Li-ion battery performances. RSC Adv. 9 (2019) 4599–4608.

[5] "Electrolytes for lithium and lithium-ion batteries" ed. T.R. Jow, K. Xu, O.Borodin, M. Ue, 2014, Springer, New York, p.384

[6] V. N. Afanas'ev, E.Y. Tyunina, M. D. Chekunova, The influence of temperature and concentration on viscous flow of solutions of Et_4NBF_4 in propylene carbonate. Russian Journal of Physical Chemistry A 83 (2009) 2069–2073.

[7] Z. Zhang, Y. Lai, J, Li , Y. Liu, Electrochemical behavior of wound supercapacitors with propylene carbonate and acetonitrile based nonaqueous electrolytes, Journal of Central South University of Technology 16 (2009) 247-252.

[8] G. Feng, J. Huang, B.G. Sumpter, V. Meunier , R. Qiao, Structure and dynamics of electrical double layers in organic electrolytes, Phys. Chem. Chem. Phys. 12 (2010) 5468–5479.

[9] J. Chmiola, C. Largeot, P.-L. Taberna, P. Simon, Y. Gogotsi, Desolvation of ions in subnanometer pores and its effect on capacitance and double-layer theory, Angew. Chem. Int. Ed. 47 (2008) 3392 –3399.

[10] Y. Zhu, S. Murali, M.D. Stoller, K.J. Ganesh, W. Cai, P.J. Ferreira, A. Pirkle, R.M. Wallace, K.A. Cychosz, M. Thommes, D. Su, E.A. Stach, R.S. Ruoff, Carbon-based supercapacitors produced by activation of graphene. Science 332 (2011) 1537–1541.
